# Evaluation of Symphony CGM, a non-invasive, transdermal continuous glucose monitoring system for use in critically ill patients

**DOI:** 10.1186/cc13629

**Published:** 2014-03-17

**Authors:** J Joseph, M Torjman, J Reich, A Furnary, S Nasraway, M McNamara-Cullinane, D Olson, D Walton

**Affiliations:** 1 Thomas Jefferson University Hospital, Philadelphia, PA, USA; 2 Cooper University Hospital, Camden, NJ, USA; 3 Tufts Medical Center, Boston, MA, USA; 4 Providence Heart and Vascular Institute, Portland, OR, USA; 5 Echo Therapeutics, Philadelphia, PA, USA

## Introduction

Glycemic control in the ICU has been shown to reduce morbidity, mortality and length of stay. However, current methods of blood glucose (BG) monitoring are invasive, intermittent and laborintensive. Continuous glucose monitoring (CGM) has potential to improve safety/efficacy of BG control. The performance of a non- invasive, transdermal CGM system (Symphony CGM; Echo Therapeutics, Philadelphia, PA, USA) was evaluated in post-surgical ICU patients.

## Methods

Adult surgical patients with planned ICU admission of ≥24 hours at four medical centers were consented. Following admission to the ICU, the skin of an upper arm was cleaned and a 6 mm diameter site was prepared with controlled micro-abrasion using the Symphony CGM system. A transdermal CGM sensor containing glucose oxidase was applied. Following a 1-hour warm-up period, a calibration was performed. Blood samples were obtained from a radial artery catheter approximately every hour, centrifuged to plasma, and glucose was measured using a YSI 2300 STAT Plus Glucose Analyzer (reference BG). A maximum of 30 reference BG samples were collected for each patient. Samples were collected as frequently as every 15 minutes for trend analysis. CGM was prospectively calibrated every 4 hours. All treatment decisions were based on BG alone. Safety was assessed by visual inspection of the site using a dermatological scale following sensor removal. A study was defined as evaluable for CGM sessions >16 hours.

## Results

Thirty-two subjects completed the study. Additional subjects were not considered evaluable due to early discharge from the ICU, failure or early removal of the radial artery catheter, or administration of intravenous acetaminophen. The study cohort was 19% female, 28% diabetes, 56% cardiac surgery, with a mean age of 65 ± 13 years. Overall mean absolute relative difference between CGM and reference BG was 12.5%. Continuous glucose error-grid analysis, which assesses point and trend accuracy, showed 98.2% of readings in the A zone (clinically accurate) and 1.2% in the B zone (benign errors). Glucose values ranged from 49 to 324 mg/dl. No device or study-related adverse events were reported.

## Conclusion

The Symphony CGM system demonstrated clinically relevant accuracy and excellent safety in a variety of patients and ICU environments. Future studies are needed to determine whether Symphony CGM can be used to direct therapy and improve BG control in this patient population.

